# Complementary and integrative medicine perspectives among veteran patients and VHA healthcare providers for the treatment of headache disorders: a qualitative study

**DOI:** 10.1186/s12906-022-03511-6

**Published:** 2022-01-25

**Authors:** Deena E. Kuruvilla, Hayley Lindsey, Amy S. Grinberg, Roberta E. Goldman, Samantha Riley, Sean Baird, Brenda T. Fenton, Jason J. Sico, Teresa M. Damush

**Affiliations:** 1 Headache Centers of Excellence Research and Evaluation Center, Veterans Health Administration, West Haven, CT USA; 2 Pain, Research, Informatics, Medical Comorbidities and Education (PRIME) Center, VA Connecticut Healthcare System, West Haven, CT USA; 3 Present Address: Westport Headache Institute, Connecticut Westport, USA; 4 Yale School of Medicine, New Haven, CT USA; 5 Department of Family Medicine, Warren Alpert Medical School of Brown University, Providence, RI USA; 6 Harvard T.H. Chan School of Public Health, Boston, MA USA; 7 Veterans Health Administration Health Services Research and Development (HSR&D) Center for Health Information and Communication (CHIC) and Quality Enhancement Research Initiative Expanding expertise Through E-health Network Development (EXTEND) QUERI) Centers, Roudebush VAMC Indianapolis, Indianapolis, IN USA; 8 Indiana University School of Medicine Indianapolis, Indianapolis, IN USA; 9 Regenstrief Institute, Inc, Indianapolis, IN USA

**Keywords:** Headache, Migraine, Complementary and integrative medicine, Veteran

## Abstract

**Objective:**

To evaluate veteran patient and provider perceptions and preferences on complementary and integrative medicine (CIM) for headache management.

**Background:**

The Veterans Health Administration (VHA) has spearheaded a Whole Health system of care focusing on CIM-based care for veteran patients. Less is known about patients’ and providers’ CIM perceptions and preferences for chronic headache management.

**Methods:**

We conducted semi-structured interviews with 20 veteran patients diagnosed with headache and 43 clinical providers, across 12 VHA Headache Centers of Excellence (HCoE), from January 2019 to March 2020. We conducted thematic and case comparative analyses.

**Results:**

Veteran patients and VHA clinical providers viewed CIM favorably for the treatment of chronic headache. Specific barriers to CIM approaches included: (1) A lack of personnel specialized in specific CIM approaches for timely access, and (2) variation in patient perceptions and responses to CIM treatment efficacy for headache management.

**Conclusion:**

Veteran patients and VHA clinical providers in this study viewed CIM favorably as a safe addition to mainstream headache treatments. Advantages to CIM include favorable adverse effect profiles and patient autonomy over the treatment. By adding more CIM providers and resources throughout the VHA, CIM modalities may be recommended more routinely in the management of veterans with headache.

**Supplementary Information:**

The online version contains supplementary material available at 10.1186/s12906-022-03511-6.

## Background

Migraine affects 1 out of 7 Americans and close to 12% of veteran patients within the Veteran Health Administration (VHA) system [1, 2]. The prevalence rates for headache substantially increase among veterans with a history of deployment-related traumatic brain injury (d-TBI) [3]. Among veterans with a history of d-TBI, 89% have the migraine phenotype [4]. While new migraine preventive and abortive treatments are emerging, research shows that a significant percentage of patients with headache are looking beyond standard medical treatments and seek to actively incorporate complementary and integrative medicine (CIM) into their individual treatment plans [5–8].

CIM is defined by the National Center for Complementary and Integrative Health (NCCIH) as treatments that are separate from mainstream medicine but may be integrated with it. NCCIH was previously called the National Center for Complementary and Alternative medicine (NCCAM) but was changed to NCCIH based on the escalation in use of complementary approaches by Americans [9]. NCCIH more accurately reflects that Americans are no longer using these approaches “alternatively” but rather in conjunction with mainstream medicine to treat a wide range of conditions and promote health. Examples of CIM are commonly divided into two main categories: Natural products (herbs, vitamins, minerals, and probiotics) and mind-body practices (yoga, chiropractic, meditation, and massage therapy). Each category is further divided into a subcategory of CIM with meditation/yoga, herbal therapies, massage/chiropractic care, and acupuncture being the top CIM approach in each category, respectively [10, 11]. While there is evidence surrounding the use of integrative approaches such as meditation/mindfulness, acupuncture, and nutraceuticals for the treatment of migraine among the general population, there are limited studies involving headache disorders in the veteran patient population specifically [12].

In 2012, the National Health Interview Survey (NHIS) reported on 88,962 American adults and found 33.2% of adults used CIM in the previous 12 months [13]. Additionally, Americans spent $30.2 billion on complementary health approaches during the same period [13]. The 2017 NHIS revealed a significant increase in CIM use since the 2012 report including yoga (from 9.5 to 14.3%), meditation (from 4.1 to 14.2%), and chiropractic care (from 9.1 to 10.3%) [14]. Retrospective studies have shown that 27% of veteran patients with chronic pain use CIM and the most common modalities used are meditation, yoga, acupuncture, chiropractic care, guided imagery, biofeedback, Tai Chi, massage, and hypnosis [15]. Prior studies have also confirmed a preference for CIM among veteran patients receiving pain management at the VHA [15, 16]. In the general population, 28–82% of people with headache disorders report using at least one of these CIM approaches, and 50% of these people do not discuss their CIM treatments with their healthcare provider [8, 10]. This data shows the need for more CIM education among healthcare providers in order to initiate the conversation with patients about which CIM approaches they have used or are interested in. This data also highlights the need for further CIM research for headache disorders based on patient preference for these modalities. With increased provider and patient education and further scientific evidence, CIM treatments can be integrated into a mainstream management plan safely.

**Fig. 1:**
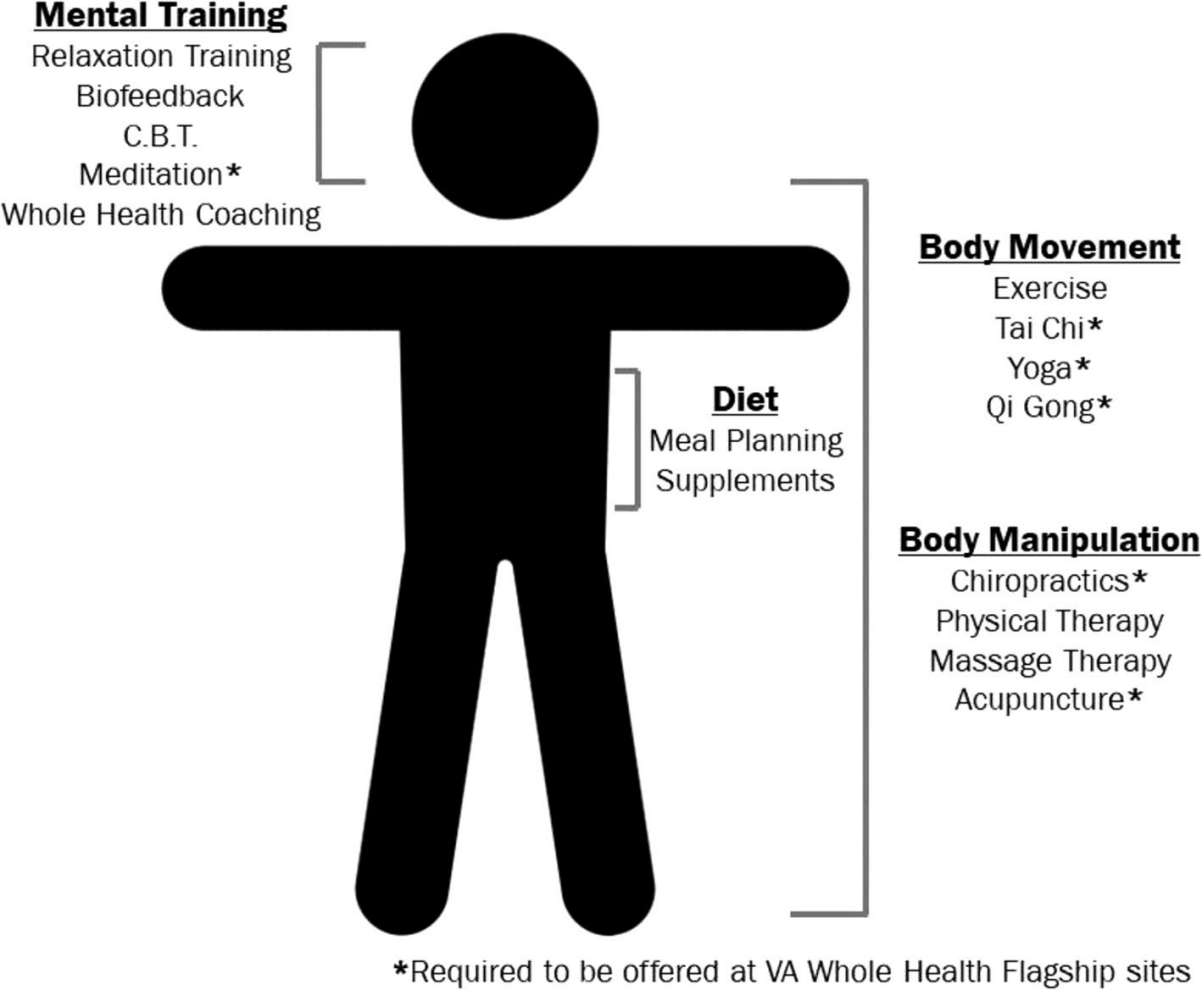
Integrative Medicine Treatments available within Whole Health Systems at Designated Veteran Health Administration Medical Centers

The VHA Headache Centers of Excellence (HCoE) initiative is a growing national program designed to improve care quality, delivery, and access for veteran patients with headache disorders to healthcare providers with headache medicine expertise and to interdisciplinary headache care clinics. HCoEs offer a breadth of therapeutic pharmacologic and non- pharmacologic modalities which are most in-line with veteran patient preferences. Prior to the inception of the HCoE program, the Whole Health initiative within the VHA health care system was instituted in specific VHA medical centers (VAMCs) to empower veteran patients to work with integrative medicine practitioners and Whole Health coaches to practice various CIM approaches such as meditation/mindfulness, yoga, exercise and several other approaches listed in Figure 1 [17–21]. The Whole Health initiative is further divided into flagship sites and design sites where flagship sites provide coaching directly to the veteran patients to promote CIM. Recognizing the value of the Whole Health program, HCoE providers have begun to refer their veteran patients with headache to Whole Health for CIM. As part of the design site program, specific elements of Whole Health are implemented in phases. Initially, these sites may have limited services offered and time availability, but as the program develops and services are expanded, flagship status can be reached [21]. The HCoE has partnered with the VHA Whole Health Initiative to implement CIM into veteran patient care plans [19]. Therefore, the overall Research Question for this qualitative study was: *What are veteran patients’ and VHA clinical providers’ perceptions of and preferences for treatment of headache with CIM modalities?*

## Methods

### Design, setting, sample and recruitment

We conducted an exploratory qualitative study to investigate how Veteran patients and VHA providers conceptualize and make use of CIM treatments for headache. Qualitative in-depth interviews provided the opportunity to collect data about this topic in participants’ own words, allowing for development of insights about how and why they hold these perceptions and why this matters to VHA headache care. We selected a purposive sample of veteran patients with the following criteria: diagnosed headache disorder; received care at HCoE; and either resolved or unresolved headache symptom management. We interviewed VHA clinical providers practicing at one of the 12 VHA HCoEs located within a Veterans Affairs Medical Center (VAMC): Cleveland, Minneapolis, Palo Alto, Richmond, San Antonio, Tampa, West Haven, Birmingham, Orlando, Pittsburgh, Salt Lake City and Greater West LA). For veteran patient interviews, clinical site leads at each HCoE were asked to identify veteran patients diagnosed with a headache disorder who received headache specialty care at the respective HCoE and either had experienced headache symptom resolution or had continuous unresolved symptoms. Veterans were not required to have experienced CIM in order to participate in this study. Clinical site leads discussed the study with veterans during clinical headache visits or phone calls. Clinical leads referred 30 veterans for participation in the study. Research staff contacted veterans in person or by phone and 20 (67%) agreed to participate. Veteran participants were unknown to interviewers prior to the study, while some provider participants were known to interviewers. For provider recruitment, the same clinical leads were contacted by email to request interviews with their key HCoE staff. Clinical site leads invited HCoE staff to participate in the study by email, during meetings, or during routine clinical operations. Fifty-three providers were invited to participate in the study, including clinical site leads, and 43 (81%) agreed to participate. Providers were not required to be CIM practitioners. Participation was voluntary and no incentives were provided.

### Standard protocol approvals, registrations, and patient consents

Institutional Review board (IRB) approval was obtained from the VHA Connecticut Healthcare System for this study, #JS0006. We obtained verbal informed consent to participate in the study and to audio record veteran patient and VHA provider interviews, which were transcribed verbatim and de-identified. Participants were told that they were being interviewed by researchers in the HCoE Research and Evaluation Center in order to inform the implementation of the HCoE program.

### Study team expertise and interview guide development

Study team members (TD, HL, JJS, AG, REG, DEK) had combined expertise in qualitative and mixed methods research, implementation science, medical anthropology, health psychology, and neurology. Two members of the study team were United Council for Neurologic Subspecialties (UCNS) board-certified headache medicine neurologists and one member was a clinical health psychologist, each of whom provides direct patient care and uses CIM modalities for the treatment of headache in VAMCs. Together, the study team used published literature and their clinical experience to develop an interview guide with open ended questions to gain veteran patient and VHA provider perspectives regarding the use of CIM in headache treatment.

### Data collection

We conducted a cross-sectional interview with participants lasting in duration 30–60 min. Interviews were conducted via telephone or at a VHA medical center in a private room by study team members (HL, RG, TD). Additional file 1 lists both provider and patient interview questions regarding the delivery and use of complimentary and integrative therapies. For providers, we asked about access of veteran patients with headache to CIH therapies and related costs; provider awareness of such therapies and suggestions for increasing awareness; and the screening and referral process. For patients, we inquired about use of complimentary and integrative therapies, how accessed, out of pocket costs, preferences, and provider communications about these therapies. Interviews were conducted until data saturation was reached based on iterative analyses by the study team. Veteran patient interviews occurred from June 2019 to November 2019, and VHA clinical providers interviews occurred from January 2019 to March 2020.

### Chart review to characterize veteran sample

We conducted a chart review to characterize our veteran sample. All available VHA Computerized Patient Record System electronic health record notes were reviewed both manually and via key word searches by a study team member (JJS) to collect information regarding veteran patient socio-demographics, headache history, concomitant mental health conditions, service-connected status, prescriptions of nutraceuticals available within the VHA (i.e., riboflavin, magnesium), and healthcare utilization (see Tables 1 and 2).

**Table 1:** Characteristics of VA Provider Participants (*N* = 38)

Discipline (N, %)	
Key Staff (n)	81
Providers Interviewed (n,%)	43, 53.09%
Neurologist (n,%)	13, 30.23%
Physiatrist (n,%)	8, 18.60%
Nurse practitioner (n,%)	5, 11.63%
Registered nurse (n,%)	3, 6.98%
Health psychologist (n,%)	3, 6.98%
Research (n,%)	2, 4.65%
Physical therapist (n,%)	1, 2.33%
Neuropsychologist (n,%)	1, 2.33%
Chiropractor (n,%)	1, 2.33%
Recreational therapist (n,%)	1, 2.33%
Program support assistant (n,%)	1, 2.33%
Anesthesiologist (n,%)	1, 2.33%
Physician Assistant (n, %)	1, 2.33%
Administrative (n, %)	1, 2.33%
Occupational Therapist (n, %)	1, 2.33%

**Table 2:** Characteristics of Veteran Participants (*n* = 20)

Sociodemographic	
Age (mean, SD)	54, 13.77
Race (n, %)	
White	15, 75%
Black	4, 20%
Asian	1, 5%
Unknown	0
Ethnicity (n, %)	
Hispanic	1, 5%
Non-Hispanic	18, 90%
Unknown	1, 5%
Gender, men (n, %)	16, 80%
TBI history (n, %)	7, 35%
Headache	
Distinct types (mean, SD)	1.4, 0.68
Migraine (n, %)	15, 75%
Tension-type (n, %)	3, 15%
Cluster headache (n, %)	1, 5%
Headache NOS (n, %)	7, 35%
PTHA (n, %)	1, 5%
Mental Health Comorbidities	
Distinct types (mean, SD)	2.05, 1.1
Depression (n, %)	10, 50%
Anxiety (n, %)	7, 35%
PTSD (n, %)	13, 65%
Dissociative disorder (n, %)	1, 5%
Adjustment disorder (n, %)	6, 30%
Panic attacks (n, %)	2, 10%
OCD (n, %)	1, 5%
Affective disorder (n, %)	1, 5%
MST (n, %)	0
None (n, %)	1, 5%
Service-Connected Status	
Total (n, %)	20, 100%. Mean service connection is 68%.
Headache (n, %)	10, 50%
Headache (mean, SD)	33%, 14.94
TBI (n, %)	2, 10%
TBI (mean, SD)	70%, 0
PTSD (n, %)	7, 35%
PTSD (mean, SD)	58.57%, 15.74
None (mean, SD)	0
Therapeutic Modalities	
Prescription abortive medication(s)	10, 50%
Prescription preventive medication(s)	17, 85%
Riboflavin	1, 5%
Magnesium oxide	3, 15%
Coenzyme Q10	1, 5%
Healthcare Utilization	
PCP only (n, %)	1, 5%
Neurology (n, %)	14, 71%
Polytrauma (n, %)	13, 65%
Health psychology (n, %)	6, 30%
Telehealth	
Pre-COVID only (n, %)	1, 5%
During COVID only (n, %)	7, 35%
Both pre- and during COVID (n, %)	8, 40%
None	13, 65%

### Qualitative analysis

Audio-recorded interviews were professionally transcribed, de-identified, and entered into NVivo 12 software [22]. In developing the codebook for qualitative coding, team members independently read and coded identical transcripts using a common codebook derived from the semi-structured interview guide. Thus, key topics asked in the interview guide were used as an initial set of codes. Subsequently, each transcript was coded by two assigned reviewers and the coded transcripts were merged into a single file where the coding team met to review and discuss similarities and differences in the coded sections, as well as agreement upon any codes that emerged during the coding process, until a shared understanding of each emergent code book item had been reached. Thematic analysis and comparative case comparisons were used to analyze coded data [23]. For thematic analysis, we identified emergent themes and patterns related to veteran patients’ and providers’ perceptions and preferences for complimentary and integrative medicine for headaches and illustrated these with direct quotations. For case comparisons, we compared the data by sites with Wholehealth flagship compared to design programs. We followed the Consolidated Criteria for Reporting Qualitative Research (COREQ) guidelines [24].

## Results

The study sample included 17 semi-structured interviews with veteran patients (20 Veteran participants total as some interviews included more than one patient) and 28 semi-structured interviews with VHA clinical providers (43 provider participants total as some interviewed included more than one provider). Interviews addressed CIM perceptions and preferences with headache care across 12 VHA HCoE sites.

### Provider characteristics

Forty-three (81%) out of 53 key staff across 12 HCoE sites were interviewed. Ten providers who were invited to participate declined or did not respond to the invitation. Most providers (30.2%) interviewed were neurologists, followed by physiatrists (18.6%), nurse practitioners (11.6%), nurses (7.0%), health psychologists (7.0%), and additional staff. Provider participants were affiliated with one of 12 different VHA HCoE sites, including Palo Alto (20.9%), Tampa (16.3%), Richmond (14.0%), San Antonio (11.6%), Cleveland (4.7%), Minneapolis (4.7%), West Haven (4.7%), Birmingham (4.7%), Orlando (4.7%), Pittsburgh (4.7%), Salt Lake City (4.7%), and West Los Angeles (4.7%).

### Veteran patient characteristics and healthcare utilization

Veteran participants (*n* = 20) comprised the sample of completed HCoE interviews from seven unique VAMC facilities, including Tampa (30.0%), West Haven (20.0%), Minneapolis (15.0%), Palo Alto (15.0%), Birmingham (10.0%), Richmond (5.0%), and San Antonio (5.0%). Eight Veterans who were invited to participate declined or did not respond to the invitation. We retrieved Veteran participant demographics and healthcare utilization data through medical chart review. Sixteen participants were male; four were female; racial/ethnic demographics of all participants were 15 White, 4 Black and 1 Asian. One participant self-reported as Hispanic; 18 self-reported as non-Hispanic and 1 was unknown. Mean age was 54 (SD = 13.77) years (Range (33–76 years). See Table 2. Our sample of veterans recorded first time VHA visits starting in 1999, with the most recent first time VHA visit recorded in 2015. First time recording of visits for VHA headache specific care was similar in range of 1999 to 2018. Seven veterans were diagnosed with a history of Traumatic Brain Injury (TBI).

On average, veteran participants were diagnosed with 2 distinct mental health comorbidities (mean = 2.05, SD = 1.10). Post-Traumatic Stress Disorder (*n* = 13) and Depression (*n* = 10) were the most prevalent comorbidities diagnosed. Anxiety (*n* = 7), Adjustment Disorder (*n* = 6), Panic Attacks (*n* = 2), Obsessive Compulsive Disorder (*n* = 1), Affective Disorder (*n* = 1) and Dissociative Disorder (*n* = 1) were also diagnosed. No comorbidities were included in the electronic health record of 1 participant.

Veterans’ diagnosed headache disorders on average included 1.4 (SD = 0.68) distinct types. Migraine was the most diagnosed headache disorder (*n* = 15). Headache NOS (Not Otherwise Specified) (*n* = 7) and Tension-type (*n* = 3) were also diagnosed among the participants. Less common headache diagnoses were Cluster Headache (*n* = 1) and Post Traumatic Headache (*n* = 1).

Among this sample of Veterans, the majority received headache care in a neurology service (*n* = 14) and a Polytrauma Center (*n* = 13). Slightly more than a quarter (*n* = 6) were seen by a health psychologist, and one was seen by primary care for headache care.

### Veteran patients’ and clinical providers’ perceptions about CIM

CIM approaches used by providers and veteran patients included the following: acupuncture, Tai Chi, chiropractic care, yoga, mindfulness/relaxation, biofeedback, cognitive behavioral therapy, massage, nutraceuticals, exercise, and cannabinoids or cannabis derivatives. According to providers and veteran patients interviewed, where Whole Health is available, CIM education and treatment has been promoted. According to both groups interviewed, health psychologists are a main driver for CIM use within these VAMCs (Fig. 1).

Four major themes emerged from interviews with both veteran patients and VHA providers: (1) CIM is useful as a self-directed or provider-driven approach which encourages empowerment of patients with headache; (2) Providers at the VHA are well versed with CIM approaches for headache care; (3) While there may be a high volume of interest from patients for CIM, there may be limited access to CIM providers to provide patient education or provider-driven CIM treatments such as acupuncture; and, (4) There is accessibility to CIM approaches with a multidisciplinary approach to headache management at many VHA facilities. Following, we discuss each of the major themes with illustrative quotations.

#### CIM is useful as a veteran self-management tool or provider-driven approach which encourages empowerment of patients with headache

Veteran patients were generally very open to CIM approaches to headache treatment. Some veteran patients stated that CIM approaches empowered them to take control over their headache treatment. Another main factor promoting the use of CIM is the veteran patient’s prior experiences with mainstream treatments. Some reported limited efficacy with prescription treatments and some had a general aversion to prescription medications and simply did not want prescription medications as part of their headache treatment plan. Some veterans also cited a fear of addiction or dependence with mainstream prescription treatments. Veteran patients and providers cited difficulties tolerating mainstream prescription treatments or having contraindications due to preexisting medical conditions.

Veteran patients highlighted the importance of being in tune with their bodies. They valued being educated on modalities they can initiate on their own and preferred this over taking prescription medications. Veteran patients also favored the Whole Health flagship model for CIM headache care to foster their own self-management for their headaches.

“Yeah, this [Whole Health] program’s been helpful, and I would recommend it to anybody. It’s one thing to give you medicine, it’s another thing to educate you because if you’re just getting a pill and not being told or educated this is what’s going on with your body and how could you be in tuned.” (101_PTFG_01_B)

VHA providers reported that since headaches can be related to the patient’s lifestyle, they prioritize education about non-pharmacological interventions. They noted that veteran patients prefer non-pharmacological interventions over mainstream medications. This patient preference is aligned with CIM therapies.

“*A lot of the veterans really prefer that [nonpharmacologic interventions]. They don’t want to take any medications*.” (103_VAPROV_01_B)

“*A lot of the patients have lots of meds already, or they are sensitive to medications, or they just don’t want to be on a lot of meds*.” (105_VAPROV_02_B)

Providers also differentiated between provider-centered CIM care and patient-centered CIM care. Providers classified acupuncture, chiropractic care and massage as provider-centered modalities and mindfulness, meditation, yoga, and Tai Chi as patient-centered modalities. With this framework, providers asserted that patients are empowered with their care if they are given education about patient-centered modalities to practice on their own or provider-centered modalities to work with someone with special training in modalities such as acupuncture, chiropractic care and massage.

“*So patient-centered care is what the focus is…and based on those, they’re able to choose some what they call provider-centered, which would be acupuncture, chiropractic, or like massage type of things versus patient centered, which would be more like mindfulness-based activities, meditation, and yoga, Tai Chi*…” (106_VAPROV_03_B)

#### Providers at the VA are well-versed with CIM approaches for headache

Veteran patients who received specialty headache care reported consistently receiving extensive education from VHA clinical providers about CIM modalities such as acupuncture and yoga at their local VHA site. This was true among veterans seen in HCoEs with and without Whole Health flagship and design sites.

“*This program [CIH] is something that they offered to me and so far, it’s been really informative. As far as whether it’s gonna work to help me with my headaches and migraines, I’m not sure. But it is a lot of information so I would say they’re doing a good job of trying to figure out ways to help me with it*.” (101_PT_02_B)

Veteran patients highlighted a point not mentioned by VHA providers. Veteran patients reported that while approaches such as acupuncture were initially helpful for them, they lost their efficacy over time. Some patients reported that CIM treatments help them cope with pain while others found CIM unhelpful.

VHA providers at Whole Health flagship and design sites claimed to have skills in CIM modalities such as acupuncture and Tai Chi. For services they could not provide themselves, they cited a referral network either within or outside of their facility.

“*Integrative health offers chiropractic [care], they offer alpha stim which can be helpful for headaches. I do battlefield acupuncture for headaches. Alot of our providers at our VA do…It kind of depends. If they [Veterans] live locally, we’ll send them to integrative health in our VA. If they’re not local…we’ll send them out for chiropractic, massage, acupuncture in the community. We do have some biofeedback and mindfulness. We have a mindfulness center as part of our Whole Health center. So, we try to tap into those*.” (111_VAPROV_02_B)

*“I 100% advocate for that [complementary and integrative medicine]. I am the Tai Chi instructor… So I have a class every Friday, and I invite all the population that they want to attend to that class, so everybody is welcome.” (101_VAPROV_03_B)*

VHA providers at the non-Whole Health sites reported routinely providing education about non-pharmacological approaches such as nutrition and supplements, and pharmacological approaches.

“*Most of the educational materials…that I provide are not necessarily headache specific but are Whole Health or integrated health related. I’ll talk about nutrition. I’ll talk about maybe supplements with them*.” (106_VAPROV_03_B)

“*We usually lay out options for the patient. I involve patients in decision-making, depending on their preferences and things that they have already tried before…so we use pharmacological and non-pharmacological*” (110_VAPROVFG_01_B)

While patients reported high interest in CIM, there may be limited providers to deliver education or provider-driven CIM treatments such as acupuncture. Veteran patients described delays in follow-up after their initial evaluation for CIM approaches such as acupuncture. Due to the growing interest in CIM approaches, veterans believed there may not be enough staff time to accommodate the volume of interest.

“*The only thing about the acupuncture with the pain clinic, [physician name] is the only one that does it, and so, if you see him today, you might see him again in*

*6 months. I mean there’s too many people and not enough time to see them all, with him*.” (108_PT_02_B)

VHA providers also described CIM popularity among patients, and noted the same problems with wait times.

“*Everybody wants massage. Everybody wants acupuncture. Then, the barrier I think for Botox is just there are so many patients that want to get it. And there’s not as many providers that can provide that. So, there is a bit of a wait sometimes for people to get in*.” (111_VAPROV_02_B)

#### There is accessibility to CIM approaches with a multidisciplinary approach to headache Management at Many VHA facilities

Veteran patients said their VA facility routinely offers CIM approaches for headache. They reported that if a veteran patient has interest, then the patient has different avenues that can be taken to be plugged in as well as a drop-in option at some centers. Veteran patients described these processes at Whole Health sites and non-Whole Health sites. VHA providers reported CIM approaches such as acupuncture, massage, psychology services such as biofeedback, and chiropractic care are readily available at their respective HCoE VA sites. This was true for Whole Health and non-Whole Health sites.

“*Every single patient gets [promotional materials] for self-management practices….Well we have something called Whole Health that we will plug into when someone has the time for more extended care. Plus, Tai Chi is done here in the facility. So, they can opt to go to it….. Like I was saying before, it was medicine, psychology, and OT. We now have a chiropractor and virtual reality and also education that started. …So when someone comes, even if they come from a distance, we’ll get those five or six appointments each time that they come. So it’s trying to get everything done at once so that they don’t have to come back and forth to get care.”* (101_VAPROV_01_B)

“*And we’re really focused on changing somebody’s lifestyle, which could have a huge impact on the headaches. We’re big here on complementary and alternative medicine. So we offer yoga. We offer Tai Chi. We have massage as well.”* (103_VAPROV_01_B)

*“It’s not directly in the pain clinic, but we have access to sending patients to integrative health and healing modalities. And that includes healing touch, mindfulness, yoga, all that. So, there’s access to send these patients if they’re interested*.” (112_VAPROV)

## Discussion

Around 8% of hospital admissions in the United States are due to adverse effects from prescription drugs [25]. Patients often express an interest in CIM due to their possible adverse effects and/or lack of efficacy [26]. The Study of CIM within the VHA is essential for this reason. Little is known about provider and patient perceptions of CIM within a VHA. In a single-site study conducted within the VHA to understand provider perspectives on CIM for patients, themes around patient awareness, preferences and access to CIM at both the provider- and facility-level were reported for the management of headaches [27]. Veteran patients and providers in that study were knowledgeable about CIM for the treatment of headache, which was true for Whole Health and non-Whole Health sites [27]. From the Veteran patient perspective, they noted that while they may have access to CIM treatments at their VHA, they have difficulty obtaining timely access and follow up for provider-driven CIM approaches such as acupuncture and chiropractic care. Veteran expectations about CIM as expressed in this study were that both initial access to and continued follow-up with providers skilled in such approaches as meditation and yoga would be requisite to meaningfully incorporating CIM into their headache management. Veteran patients also reported that CIM treatments have variable efficacy for the treatment of headache disorders.

From a VHA provider perspective, they reported recommending several CIM modalities to patients for the management of chronic headache disorders within the VHA system. This was especially true for patients for whom mainstream headache therapies were ineffective or had undesirable side effects [26]. Providers within VHA hospital systems with more robust CIM and Whole Health Initiative infrastructure generally expressed better awareness of the evidence behind CIM for headache, and knowledge of local resources and referral protocols. While VHA providers without similar infrastructure also encouraged incorporating CIM into treatment regimens, they cited access as a barrier to recommending CIM to their patients. The VHA approaches headache treatment from a multidisciplinary standpoint according to both veteran patients and VHA providers in Whole Health and non-Whole Health systems. A multidisciplinary approach is especially important for the treatment of headache as many patients have comorbidities such as depression, anxiety, and musculoskeletal pathology that if addressed could result in better pain outcomes [28, 29].Well known headache centers across the United States often have headache specialist physicians and nursing staff to provide teaching and education, psychology, psychiatry, and physical therapy amongst other specialties [28].

Outside of the headache literature, patients have reported preferences for integrative treatments because of prior experience with and dissatisfaction surrounding the use of mainstream therapies, a sense of autonomy when using integrative treatments, and integrative approaches aligning more with their personal beliefs about overall health and wellness [30]. Medication adherence to preventive medications is notoriously poor given side effects profiles (e.g. cognitive slowing, weight gain, teratogenicity) and dosing regimens which range from once to thrice daily [31]. Among patients with chronic migraine, adherence to medications ranges from 26 to 29% at 6 months and decreased further to 17 to 20% at 12 months [32]. Studies have shown that about 66% of patients recommended for various CIM approaches followed through with appointment with their CIM provider [33].

Studies show that the elements of a successful CIM program include: strategic planning, enthusiastic CIM leaders, leadership support, positive attitudes towards CIM by providers, patient attitude perception, evidence-based CIM, champions and effective marketing [34]. Challenges to implementing CIM are difficult with hiring, insufficient funding, patient access, difficulties in coding/documenting CIM use, insufficient space, staff and time and appropriate environments [35]. The VHA Whole Health initiative embraces many of these elements and incorporated them into HCoEs through Whole Health programs, as evidenced in our study by differences and education about the utility of CIM and headache management and increased availability of referrals between HCoEs with and without Whole Health programs.

### Strengths and limitations

Major strengths of this study include interviews conducted with a diverse group of veteran patients with headache in terms of race/ethnicity and headache type and with providers across disciplines. Interviews were also conducted in geographically dispersed sites. This sample of participants only included those who received or delivered care within the VHA health system. For this reason, the perspectives in this study may not be the perspectives of the general VHA population where CIM is less widely available. Furthermore, the veteran patient sample largely consisted of men. A unique opportunity remains to conduct a study with more women to learn about CIM preferences within headache for a population more prone to this neurological condition. Neither veteran patients nor healthcare providers mentioned either the level of evidence for CIM in headache or out of pocket costs. According to the 2007 National Health Interview Survey, people spent 14.9 billion dollars on CIM approaches for the treatment of various pain conditions [35]. These expenses were purely out of pocket because most insurers do not cover CIM treatments outside of the VHA system [35]. The VHA has a different reimbursement system, which makes this large healthcare system a unique setting to understand the utility of CIM where patients have a different health coverage structure which largely covers these treatments.

## Conclusions

Many veteran patients use complementary and integrative medicine because these modalities fit in with their health therapeutic preferences, promote self-management, and resonate with their values about headache treatment. The VHA has a Whole Health system which promotes adjuvant complimentary and integrative medicine to traditional therapies to service its veteran patients with chronic headache disorders [17]. Addressing veteran patient preference for CIM will ultimately enhance relevant, effective and desired care for this population. Gaps in the current knowledge base include treatment trials on specific CIM modalities for the treatment of specific headache disorders such as post-traumatic headache and migraine and education about CIM approaches among VHA providers. It may be beneficial to expand CIM services uniformly across the VHA network in the United States.

## Supplementary Information

**Additional file 1.** Interview Guide Questions.

## Data Availability

The data used and analyzed during this current study are not publicly available because they must remain on the Department of Veteran Affairs (VA) servers. A limited de-identified data set may be available upon request and fulfillment of the VA data use agreements.

## Abbreviations

CIM: Complementary and Integrative Medicine
VHA: Veterans Health Administration
HCoE: Headache Centers of Excellence
NCCIH: National Center for Complementary and Integrative Health
VAMCs: VA Medical Centers
TBI: Traumatic Brain Injury
