# Supplementary material for: Complementary and integrative medicine perspectives among veteran patients and VHA healthcare providers for the treatment of headache disorders: a qualitative study
Source: BMC Complement Med Ther. 2022 Jan 25;22:22. doi: 10.1186/s12906-022-03511-6 (PMC8790919; doi:10.1186/s12906-022-03511-6)
Supplement: Supplementary file 1 — Additional file 1. Interview Guide Questions. [file 12906_2022_3511_MOESM1_ESM.docx]

**Appendix 1**. Interview Guide Questions.

1. Providers: We know from previous interviews with Veterans with Headache disorders that they desire nonpharmaceutical therapies – Chiropractic, acupuncture, Tai Chi, yoga, biofeedback, etc. How would a Veteran with headache access such services at your VAMC?
   1. Does the Veteran need to initiate or do you have a screening and prescription mechanism? If yes, what is it? How does it work?
   2. Are these services offered within the HCOE or are they part of the larger VAMC?
   3. How much does it cost the Veteran? Is this a barrier to use?
   4. What do you think is the best strategy to integrate complimentary integrative health for Veterans with headache so that Veterans can readily access such services?
   5. Do you think your clinical providers are aware of the CIH services and offer to their Veteran patients with headache? Why or why not?
   6. What can be done to make clinicians more aware of these services and provide access to their patients?
   7. Do you collaborate/communicate with your facility’s CIH (complementary integrative health) coordinator?
2. Veterans: What nonpharmaceutical therapies do you use? [PROMPTS: CIH muscle relaxation exercises (meditation, yoga)?]
   1. How did you hear about these therapies? [PROMPTS: Did a VA staff member invite you to try these therapies? Other? What made you decide to try these therapies? Maintain?]
   2. What do you like the most about complementary and integrative health [alternative therapies to traditional pharmaceutical therapies and rehabilitation, Yoga, Tai Chi, meditation]? The least?
   3. How do you suggest VA clinicians offer these complimentary integrative therapies to a Veteran who is currently not using? Ideally where should these therapies be offered to best reach Veterans?
   4. Do you wish your physician would offer more complimentary integrative therapies? Why or why not?
   5. Do you have to pay out of pocket for any of these therapies?
